# Our Hospitals as Patients Find Them

**Published:** 1887-10-08

**Authors:** 


### OUR HOSPITALS AS PATIENTS FIND THEM.

A Country Surgeon writes :—"Your valuable paper being the recognised channel for communication relating to hospital management, I shall be obliged if you will kindly give me a small space in order to call the attention of managers and governors to a few points which I submit for their consideration.

"(1) *The Visits of Friends.*—At some of our metropolitan hospitals the regulations as to the visits of relatives and friends are adhered to with strictness, and I submit that the hours fixed are those when many of the poorer classes are so much engaged, that frequently a poor man, and sometimes his wife, is unable to obtain leave of absence from his employment, and therefore cannot see his daughter or son for weeks together. Would it not be practicable in such cases for the house-surgeon to give a special admission at an hour convenient to the hospital staff and to the relative?

"(2) *As to Diet.*—For many years it has appeared to me that the 'Mede and Persian' law is too much applied in our London hospitals in the item of diet. In country hospitals, especially those called 'Cottage' hospitals, considerable latitude is allowed the matron and nurse, and the appetites of the patients are duly considered in the provision made for their meals and also for the little intermediate 'nossets,' for which the patient is most grateful, and which frequently are more relished by the sick than a regular repast of which all partake. It will probably be said that where the patients are few the difficulty does not arise which constitutes a serious obstacle when they are numerous: and this no doubt is true; but even in our largest hospitals a great deal may be effected in this way. To a country man or woman who is in the habit of taking intermediate meals, the strict hours of hospital feeding become irksome. They probably have been accustomed to eat at 11 a.m., and have something between dinner and tea, but this is forbidden in most large hospitals. Surely some discretionary power could be entrusted to the nurse to give a cup of beef-tea or gruel, or a slice of bread-and-butter and a cup of cocoa if desired. The time occupied would be short, and at 11 a.m. and about 3 or 4 p.m. work in the wards is not heavy.

"But a more weighty complaint is made. Everyone accustomed to illness knows that very frequently sick people, in whatever rank of life, require a constant change of food in order to relish it sufficiently to enable them to take enough to benefit them properly, but I regret to say that in many hospitals this variety of food is altogether wanting. I give an instance. At one of our large London hospitals I lately visited a patient. She had been an inmate many weeks. At first she could eat 'slops' only, but instead of the varieties she had been accustomed to, and which are so readily obtainable, there was the same 'porridge' day after day, varied by bread and milk once a day. Then she was ordered 'meat' diet, and this consisted of a 'chop' every day without any exception. At a suggestion the surgeon ordered her 'fish' alternately, and then 'chop' divided the honours with 'sole,' and for three weeks no other meat, no other fish was given. Surely this is not right. Why cannot varieties of meat be given? Roast and boiled mutton, roast beef, beefsteaks, hashed beef and mutton, rabbits, are all as cheap and as easily procurable as mutton for 'chops'; and whiting, cod, and haddock are far more plentiful and less costly than 'sole.' Then again milk puddings in great variety are a cheap, very wholesome, and excellent adjunct, either at dinner or supper, especially for the latter if cold. I plead therefore for the sick poor in our hospitals that they may have a greater variety in their food, certain as I am that it will conduce to their comfort and to their more speedy convalescence.

"One more suggestion is frequently made by persons who are acquainted with the circumstances of those who constitute a large proportion of hospital inmates. In some of our hospitals, tea, sugar, and butter are among the articles of diet, but in others the patient must pay extra for these, or even provide them; but surely it is not reasonable that these necessaries (for they are such) should be omitted in any hospital dietary, and many patients have to go without because they have not the means to purchase them. Is this right? Some, too, of our hospitals allow tea, but if the patient does not like tea and wishes to have cocoa, he must provide it himself; but it appears to me that there is no valid reason why both should not be allowed. I think another great principle in hospital treatment underlies these matters. I contend that it is most undesirable to allow the introduction of any article of diet into a hospital other than those provided by the hospital itself. I know that improper food and drink are continually being surreptitiously smuggled into a hospital, and while condemning this most strongly, I hold that the authorities themselves are chiefly to blame, because the internal administration ought to provide everything desirable and necessary for the patients resident therein; and if this were done a strict prohibition could be issued, and would in such case be sufficient to prevent the existing evils. Our hospitals are real charities. Why, then, allow such a small matter as a want of variety in diet to militate against a patient's recovery? Or why, for the sake of a few hundred pounds yearly, call upon the unfortunate sick and often destitute inmates to purchase necessaries such as tea, cocoa, butter, and sugar? I cannot believe that many of the benevolent donors to the funds of our hospitals are aware of this illiberality, and I do not doubt that if their attention is called to it they will readily increase their donations so as to make the whole system of hospital treatment more efficacious, more valuable, and more complete, by insisting that everything requisite for the patients shall be supplied to them gratuitously while they are inmates of these most valuable institutions."

